# Adding the third dimension: 3D convolutional neural network diagnosis of temporal lobe epilepsy

**DOI:** 10.1093/braincomms/fcae346

**Published:** 2024-10-10

**Authors:** Erik Kaestner, Reihaneh Hassanzadeh, Ezequiel Gleichgerrcht, Kyle Hasenstab, Rebecca W Roth, Allen Chang, Theodor Rüber, Kathryn A Davis, Patricia Dugan, Ruben Kuzniecky, Julius Fridriksson, Alexandra Parashos, Anto I Bagić, Daniel L Drane, Simon S Keller, Vince D Calhoun, Anees Abrol, Leonardo Bonilha, Carrie R McDonald

**Affiliations:** Department of Radiation Medicine and Applied Sciences, University of California San Diego, San Diego, CA 92037, USA; Electrical & Computer Engineering, Georgia Institute of Technology, Atlanta, GA 30332, USA; Department of Neurology, Emory University, Atlanta, GA 30322, USA; Department of Mathematics and Statistics, San Diego State University, San Diego, CA 92115, USA; Department of Neurology, Emory University, Atlanta, GA 30322, USA; Department of Neurology, Medical University of South Carolina, Charleston, SC 29425, USA; Department of Epileptology, University Hospital Bonn, Bonn 53127, Germany; Department of Neuroradiology, University Hospital Bonn, Bonn 53127, Germany; Department of Neurology, University of Pennsylvania, Philadelphia, PA 19104, USA; Department of Neurology, NYU Langone Medical Centre, New York City, NY 10016, USA; Department of Neurology, School of Medicine at Hofstra/Northwell, Hempstead, NY 11549, USA; Department of Communication Sciences and Disorders, University of South Carolina, Columbia, SC 29208, USA; Department of Neurology, Medical University of South Carolina, Charleston, SC 29425, USA; Department of Neurology, University of Pittsburgh, Pittsburgh, PA 15261, USA; Department of Neurology, Emory University, Atlanta, GA 30322, USA; Department of Pharmacology and Therapeutics, University of Liverpool, Liverpool L9 7LJ, UK; Center for Translational Research in Neuroimaging and Data Science on Systems, Atlanta, GA 30303, USA; Center for Translational Research in Neuroimaging and Data Science on Systems, Atlanta, GA 30303, USA; Department of Neurology, Emory University, Atlanta, GA 30322, USA; Department of Radiation Medicine and Applied Sciences, University of California San Diego, San Diego, CA 92037, USA

**Keywords:** temporal lobe epilepsy, structural neuroimaging, convolutional neural network, MRI-negative epilepsy, machine learning

## Abstract

Convolutional neural networks (CNN) show great promise for translating decades of research on structural abnormalities in temporal lobe epilepsy into clinical practice. Three-dimensional CNNs typically outperform two-dimensional CNNs in medical imaging. Here we explore for the first time whether a three-dimensional CNN outperforms a two-dimensional CNN for identifying temporal lobe epilepsy-specific features on MRI. Using 1178 T1-weighted images (589 temporal lobe epilepsy, 589 healthy controls) from 12 surgical centres, we trained 3D and 2D CNNs for temporal lobe epilepsy versus healthy control classification, using feature visualization to identify important regions. The 3D CNN was compared to the 2D model and to a randomized model (comparison to chance). Further, we explored the effect of sample size with subsampling, examined model performance based on single-subject clinical characteristics, and tested the impact of image harmonization on model performance. Across 50 datapoints (10 runs with 5-folds each) the 3D CNN median accuracy was 86.4% (35.3% above chance) and the median *F*1-score was 86.1% (33.3% above chance). The 3D model yielded higher accuracy compared to the 2D model on 84% of datapoints (median 2D accuracy, 83.0%), a significant outperformance for the 3D model (binomial test: *P* < 0.001). This advantage of the 3D model was only apparent at the highest sample size. Saliency maps exhibited the importance of medial–ventral temporal, cerebellar, and midline subcortical regions across both models for classification. However, the 3D model had higher salience in the most important regions, the ventral-medial temporal and midline subcortical regions. Importantly, the model achieved high accuracy (82% accuracy) even in patients without MRI-identifiable hippocampal sclerosis. Finally, applying ComBat for harmonization did not improve performance. These findings highlight the value of 3D CNNs for identifying subtle structural abnormalities on MRI, especially in patients without clinically identified temporal lobe epilepsy lesions. Our findings also reveal that the advantage of 3D CNNs relies on large sample sizes for model training.

## Teaser

Kaestner *et al*. report that convolutional neural networks using whole-brain three-dimensional T1-weighted scans can detect the neural signature of epilepsy and outperforms a two-dimensional convolutional neural network model. Performance is particularly encouraging for scans judged by neuroradiologists to be non-lesional.

## Graphical Abstract

**Graphical Abstract:**
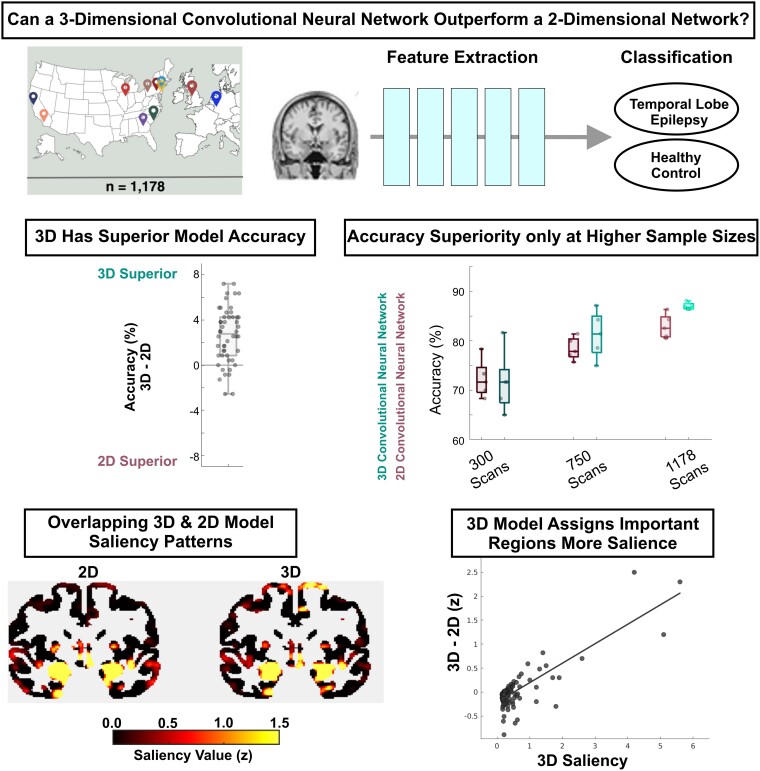

## Introduction

Medical assistance tools powered by artificial intelligence (AI) promise to increase human efficiency and accuracy.^1-3^ A potential application is the identification of temporal lobe epilepsy (TLE) from structural neuroimaging.^4,5^ Patients with visually identified hippocampal sclerosis (HS; i.e. MRI-positive patients) are more likely to have a well-localized seizure focus and improved surgical outcomes compared to patients with no MRI-identified lesion.^6^ However, many TLE patients (30–50%) have no discernable TLE lesions on MRI (MRI-negative patients)^7,8^ despite up to 50% of patients labelled as MRI-negative showing subtle HS on histopathology.^9^ Because the lack of identifiable lesions on MRI introduces uncertainty in clinical decision-making and may delay treatment, tools to enhance radiological human detection of a non-invasive TLE neural signature could greatly benefit treatment because surgical outcomes in MRI-negative patients can match MRI-positive patients.^8^

Several decades of quantitative imaging research in TLE have identified widespread neural pathology beyond the hippocampus.^10-13^ These pathological changes are detectable using structural T1-weighted scans, with the pattern being subtle but consistent across patients.^14^ In small cohorts, this pathology has been associated with TLE disease onset,^15^ disease duration,^16^ seizure onset localization,^13^ and clinical outcomes such as surgical outcome,^17^ and cognitive impairment.^18^ Pioneering tools such as NeuroQuant^19,20^ have leveraged hippocampal structure for use by clinicians. Indeed, the majority of machine-learning research in epilepsy has focused on extracting pre-defined structural features of interest for use with approaches such as support vector machines.^21-26^ Therefore, in both clinical practice and research, the use of structural neuroimaging in TLE remains mainly focused on discrete structures, neglecting the potential of an overall pattern of TLE pathology.

Convolutional neural networks (CNN) present a path towards incorporating these patterns into clinical practice. 2D CNN models can assist with the diagnosis^27^ and lateralization of TLE,^28^ differentiation of TLE and Alzheimer’s disease,^29^ and the detection of HS.^30^ Maps revealing the regions most important to classification (i.e. saliency maps) demonstrate that CNN models leverage the widespread pathological neural signature of TLE. However, 2D models are blind to depthwise spatial relationships in one direction and may not capture important TLE-specific features. 3D CNNs can learn volumetric images without the need for slicing, theoretically preventing significant loss of information. This has proven advantageous in tasks as diverse as lung^31^ and prostrate^32^ cancer screening, brain age prediction^33^ and Alzheimer’s detection.^34^ Because TLE pathology is often in regions in which volumetry is key, we hypothesize that a 3D CNN can leverage spatial relationships across the entire volume to further improve performance. This is now possible due to the availability of high-powered computers and large datasets that mitigate the problem of overfitting.^35^ Therefore, 3D CNNs could represent a more powerful tool for detecting the full TLE neural signature and translating knowledge of brain pathology in TLE into a clinical assistance tool to aid clinicians in detecting epilepsy-related lesions.

Here, we advance earlier work by applying a 3D CNN in an international cohort of 12 surgical centres totalling 1178 T1-weighted scans, larger and more diverse than previous CNN samples in epilepsy (*N* range from 157 to 359). We compare 3D CNN to 2D CNN models, test the impact of sample size and image harmonization on model performance, examine performance at the individual patient level, and characterize how knowledge of 3D versus 2D spatial relationships changes in CNN saliency maps.

## Materials and methods

### Participants

#### Data sources

589 participants with TLE and 589 HCs were derived from 12 different sites: the Medical University of South Carolina, Emory University, New York University, Northwell University, University of Bonn, University of Pittsburgh, University of Pennsylvania, Rush University, University of California San Diego, University of California San Francisco, University of Liverpool, and the Human Connectome Project. Patients were recruited if they met the following criteria: (1) a diagnosis of drug-resistant unilateral TLE by the treating clinical team based on a combination of clinical, neurophysiological, and radiographic findings in accordance with the International League Against Epilepsy.^36^ Patients were excluded if they had MRI-identified lesions other than HS (e.g. tumours, vascular malformations) or an identified extratemporal focus. Frequently, additional neuroimaging supplemented the evidence favouring medial temporal ictal onset, particularly PET and SISCOM SPECT imaging. Clinical and demographic variables were collected for research when available. Institutional Review Board approval was obtained through the Institutional Review Board where participants were enrolled. All relevant ethical regulations were followed, and informed consent was obtained at the respective facilities.

#### Sample

We obtained characteristics for each patient from each centre when available (Table 1). Centres varied with regard to readily available demographic and clinical variables, which contributed to variability in the total number of datapoints available. Supplementary Table 1 includes variables divided by site.

**Table 1:** Participant information

	HC	TLE	Stats
N	589	589	–
Age	37.4 (14)	38 (12)	*t*(1070)=−0.682; *P* = 0.5
Sex (F/M)	266/323	223/306	FET = 1.13; *P* = 0.33
Education	15.6 (3.4)	13.5 (2.4)	*t*(390) = 8.26; *P* < 0.001
Seizure onset (L/R)	–	268/227	–
Age of seizure onset	–	19.4 (13)	–
Disease duration	–	18.6 (14)	–
HS (no/yes)		244/233	

Categorical variables are listed as *N*. Continuous variables are listed as mean (standard deviation).

HC = healthy control; TLE = temporal lobe epilepsy; F = female; M = male; L = left; R = right; HS = hippocampal sclerosis.

Centres varied with regard to readily available demographic variables, which contributed to variability in the total number of data points available per variable.

### Image acquisition

Scanner type and acquisition parameters varied across institutions. Supplementary Table 2 includes parameters divided by site.

### Imaging pre-processing

T1-weighted MRI images were preprocessed using Nii_preprocess (https://github.com/neurolabusc/nii_preprocess/) using SPM12 (version 7771; http://www.fil.ion.ucl.ac.uk/spm/software/spm12/) and CAT12 (version 2000; http://www.neuro.uni-jena.de/cat12/).

#### Image processing pipeline

The pipeline included from the Statistical Parametric Mapping (SPM) with the CAT12 extension^37^: normalization, tissue segmentation, smoothing/thresholding, slice extraction, and labelling. We normalized all T1-weighted images into standard stereotaxic MNI152 space (113 × 137 × 113) using the SPM normalize function with the following parameters: bias regularization = 0.0001, bias full width at half maximum = 60, tissue probability map = TPM.nii, voxel size = 1 × 1 × 1 mm3, and fourth-degree b-spline interpolation. We smoothed images using SPM’s smooth function (a three-dimensional FWHM, 10 mm). The smoothing was performed to minimize individual variability in sulci and gyri positioning.

#### Harmonization across sites

We applied ComBat^38^ to test whether image harmonizing improved performance using https://github.com/rpomponio/neuroHarmonize^39^ to remove the site-specific bias from the training, validation, and test datasets. Accordingly, we first fit the harmonization model’s parameters on the training data and then applied the model to the validation and test data. It is important to optimize a harmonization model only by using the training samples to avoid potential information leakage, especially in machine-learning pipelines. If harmonization happens on the entire data it may lead to false overestimations of performance, originating from knowledge learned from out-of-training samples.^40^

### Artificial neural network

#### Architecture

We built an FCNet model inspired by Peng *et al*.^41^ for classifying TLE versus HC. FCNet was chosen after a comparison between FCNet, Resnet, and Alexnet. We trained 3D ResNet 18 on one iteration of 5-fold cross-validation, achieving an average accuracy of 81.63%. We also trained a 3D AlexNet, which shares the same architecture as the FCNet but replaces the last average layer with fully connected layers, resulting in an accuracy of 84.46%. Finally, we modified the AlexNet by replacing the fully connected layers with an average pooling layer to create a fully convolutional network (i.e. FCNet), which achieved an accuracy of 86.72%. Based on these preliminary results, we selected the FCNet for further evaluation with nine additional iterations of 5-fold cross-validation.

**Figure 1:**
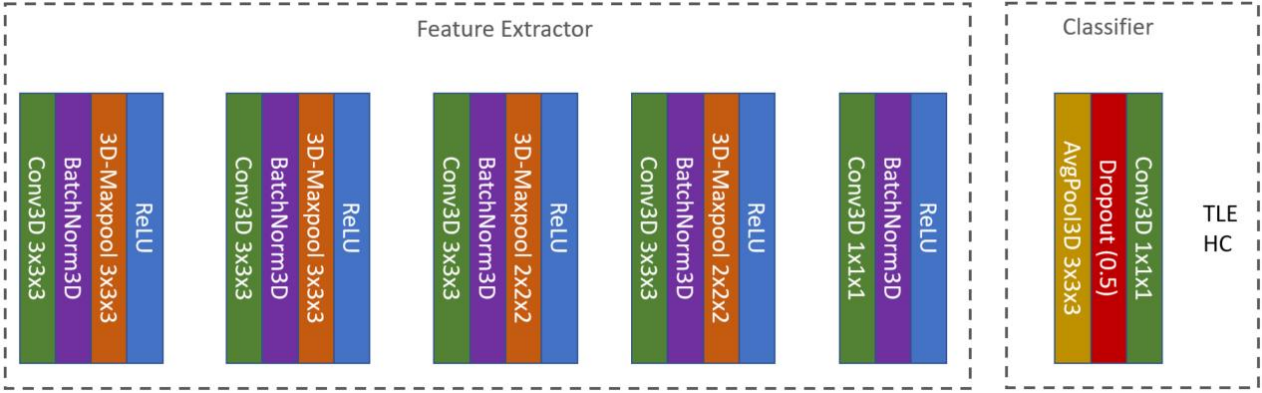
**Model architecture.** A fully 3D Feature Extractor consisted of whole-brain input into a five-layer convolutional neural network (CNN). The Feature extractor contained four Convolution-BatchNorm3D-MaxPool-ReLu layers and a fifth Convolution-BatchNorm3D–ReLu layer. The Feature Extractor was followed by a Classifier with two outputs, one for healthy controls (HC), and one for temporal lobe epilepsy (TLE).

The architecture is depicted in Fig. 1, comprising a feature extractor and classifier. The feature extractor contains four sequential convolutional layers, each accompanied by batch normalization, max pooling, and ReLU activation. An additional convolutional layer follows, complete with batch normalization and ReLU activation. The classifier consists of an average pooling layer, a dropout layer with a 0.5 dropout probability, and a 1 × 1 × 1 convolutional layer with an output dimension of 2. Kernel sizes are specified in the figure.

For comparison, we also implemented a 2D version of the FCNet, where all convolution and pooling kernels operate in 2D space. To accommodate the 3D input images, we treated each coronal slice of the 3D images as a single channel, resulting in 113 input channels for the first layer. Finally, we implemented a shuffled-CNN model to benchmark chance, identical to the 3D CNN model but labels in the training set were randomly shuffled while labels in the test set were maintained.

#### Model execution

We employed stratified 5-fold cross-validation (S5-CV), repeated 10 times, to divide the data into training and test sets, ensuring a consistent distribution of classes across all folds. A balanced dataset was used to mitigate potential biases caused by the majority class (i.e. the number of HC images was selected to match the number of TLE images) while maintaining a closely matched age and sex distribution. Each 5-fold cross-validation run comprised 1-fold as a test sample and the remaining 4-folds as training/validation samples. This training/validation portion of the data was divided into training and validation sets at an 80:20 ratio. In every S5-CV iteration, the model was trained on the training set, and the best model was identified based on the validation set (refer to the Hyperparameter Tuning section for further details). Finally, the selected model was applied to the test data, which it was completely blind to during training.

#### Hyperparameter tuning

When training our model we utilized the grid search technique. Supplementary Table 3 presents the hyperparameters and their corresponding value sets employed in this study. All models underwent training for 400 epochs, and early stopping with train patience of 40 epochs was incorporated to counteract overfitting. We trained the models using two prominent optimizers, stochastic gradient descent and Adam, with a learning rate ranging from 0.00001 to 0.1, employing a multiplication step of 0.1. We also experimented with various input channel sizes for the convolutional layers. 3D and 2D CNN models were each allowed to find their own hyperparameter space on each run, but each was restricted to the same search space.

### Model evaluation

#### Performance metrics

Each model was characterized across 50 iterations (10 runs with 5-folds) with two main measures of performance: accuracy and *F*1-score. All measures were derived from the final model performance on the left-out test dataset. The *F*1-score combines precision (also called ‘positive predictive value’) and recall (also called ‘sensitivity’) into a single measure via the harmonic mean. It complements accuracy and is most important when classes are imbalanced, which is not a concern here due to our study using balanced classes. For completeness, we include other commonly reported metrics in our tables including sensitivity, specificity, positive predictive value, and negative predictive value. We derived median performance and performance variability using the interquartile range (IQR). We did not use the area under the curve because it is equivalent to accuracy when two classes of equal size are used.

#### Model comparison

To compare our 3D CNN model (unharmonized) to the other three models (3D-shuffled, 2D, 3D-harmonized), we employed the frequency distribution comparison index (FDCI) value [(# of times 3D CNN Accuracy > Alternative Model Accuracy/total # of Models)*100 for a percentage]. We also visualize subtractions from each of the 50 iterations Accuracy and *F*1 score for the 3D CNN versus alternative models in our figures.

## Feature visualization

We employed a vanilla backpropagation method^42^ to create saliency maps, which highlight how strongly voxels contributed to the classification task. This process involves conducting a forward pass through the model, calculating one-hot encodings for each target class (HC/TLE), and executing a backward pass concerning the one-hot encoding. This yields the input image gradients in relation to the output class, which are subsequently presented as the image's saliency maps. Therefore, voxels that contribute the most to the classifier have higher saliency values. It is important to note that the final model of each iteration is utilized to compute the saliency maps for the associated test set samples. Therefore, with 5-fold cross-validation we averaged across the five-folds for each model which gave us 10 values for each voxel to ensure stability of measured salience. Saliency values were *z*-scored for ease of interpretation. We used the automated anatomical labelling (AAL) atlas^43^ to derive average region of interest (ROI) saliency values.

## Single participant performance

Finally, we examined if the CNN model’s performance at the single-patient level varied based on clinical features. Each time a patient or HC was in the test group, we tabulated whether they were successfully classified. Therefore, each patient received an accuracy score from 0 to 100%. To compare these to patient features, for categorical features we performed a *t*-test between subgroups (e.g. female accuracy versus male accuracy). For numerical features, we performed a correlation between the clinical feature and patient accuracy (e.g. age correlated with accuracy).

## Statistical analysis

HC versus TLE characteristics: One-way analysis of variance (ANOVA) for continuous variables or Fisher’s exact tests for categorical variables examined differences among HC and TLE.

Model comparison: We assessed significant differences in model performance in two ways. First, a binomial test was employed to determine if FDCI was significantly different from chance (i.e. if a model had significantly superior performance relative to its FDCI ‘competitor’). This was done by comparing the actual number of ‘wins’ to a random chance of 50% (e.g. across 50 runs, chance would suggest each model would ‘win’ 25 times). Second, we used a Wilcoxon sign-rank test to directly compare the 50 obtained accuracies between the two models.

Saliency Correlations: To compare saliency value correlation across regions between 3D and 2D CNN, we used Spearman’s rho.

Single-Subject Performance: To characterize the effect of patient characteristics on 3D CNN model performance, for continuous variables we assessed correlation with individual subject performance using a spearman’s rho. For categorical variables, we ran a one-way ANOVA with individual subject performance as the dependent variable.

## Results

### Patient demographics

#### HC versus TLE

Table 1 contains statistics between HC and TLE demographic variables and descriptive statistics for TLE clinical variables. No significant differences were found in age or sex proportion (both *P*_s_ > 0.05) between the groups. However, HCs had more years of education (*P* < 0.001).

### Versus 3D-Shuffled versus 2D model performance

#### Model evaluations

**Figure 2:**
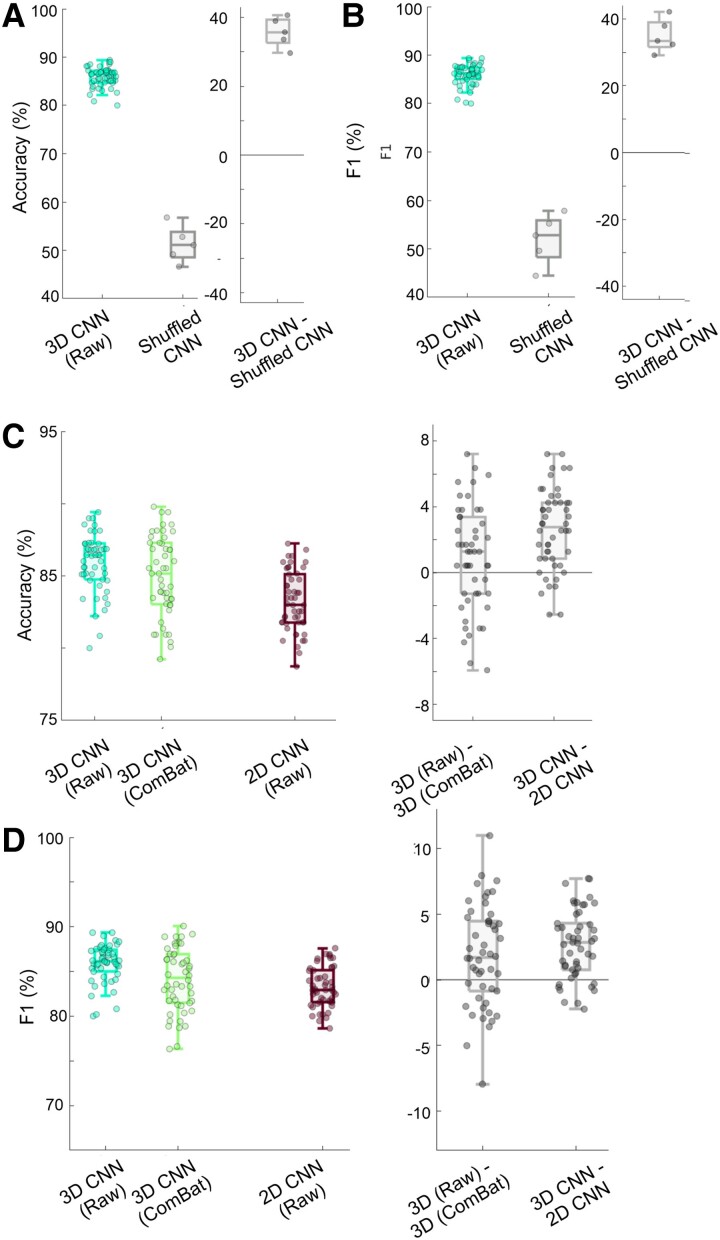
**Model performance.** Each datapoint in the scatterplots represents one of the 5-folds from one of the 10 runs for each model (for a total of 50 datapoints). (**A**) Accuracy for the 3D convolutional neural network (3D CNN; left side of plot) compared to the shuffled-CNN (right side of plot) and subtraction plot across runs. (**B**) *F*1-score for the 3D CNN and Shuffled-CNN and subtraction plot across runs. (**C**) Accuracy for the 3D CNN on the raw data (left side of plot) compared to the ComBat harmonized 3D CNN (middle of plot) and 2D CNN (right side of plot) as well as the subtraction plot across runs (3D CNN minus other model). The 3D CNN outperformed the 2D CNN on 84% of runs, which represented a significant improvement (binomial test: *P* < 0.001). Further, directly comparing accuracies via the Wilcoxon sign-ranked test revealed the same results (*P* < 0.001). Both these statistical tests apply to the left plot, between the 3D CNN versus the 2D CNN datapoints. (**D**) *F*1-score for the 3D CNN on raw data, 3D CNN on ComBat harmonized data, and 2D CNN as well as the subtraction plot across runs (3D CNN minus other model).

The 3D and 2D models were trained across 10 runs with a 5-fold structure, yielding 50 iterations (i.e. datapoints). Table 2 contains the FDCI and median values of model performance, Fig. 2A-B illustrates model performance relative to the shuffled-CNN model (i.e. data-defined chance), and Fig. 2C-D illustrates model performance relative to the 2D model. Overall, the 3D model had a median accuracy of 86.4% (IQR: 2.5%) and a median *F*1 score of 86.1% (IQR: 2.5%). To ensure that our balanced sample leads to the actual chance being ∼50%, we also ran 1 model of shuffled-CNN with a 5-fold structure giving us five iterations. The shuffled-CNN model had a median accuracy of 51.1% (IQR: 5.3%) and a median *F*1 score of 52.8% (IQR: 7.6%). Therefore, our model accuracy outperformed chance by 35.3% (i.e. ∼414 additional patients/controls correctly identified). In comparison to the 2D model, the 3D model had an FDCI score of 84%, which represented a significant improvement over the 2D model (binomial test: *P* < 0.001). The FDCI reflects how often one model had superior performance to the other model on the same stratified sample. Directly comparing accuracies via the Wilcoxon sign-ranked test revealed the same results (*P* < 0.001). Overall, the 2D model had a median accuracy of 83% (IQR: 3.3%) and a median *F*1 score of 82.9% (IQR: 3.5%). We note that the 3D CNN model accuracy outperformed the 2D model by a median accuracy of 3.4% (i.e. 39 additional TLE/HC correctly identified).

**Table 2:** Model performance

		Accuracy	Sensitivity	Specificity	PPV	NPV	F1
**3D Model (raw)**	Median	86.4 (2.5)	86.4 (5.8)	85.6 (6.8)	86.1 (5.1)	86.1 (4.3)	86.1 (2.5)
**3D Model (shuffled)**	Median	51.1 (5.3)	52.5 (10.9)	53.0 (16.9)	51.1 (5.1)	51.0 (5.5)	52.8 (7.6)
**2D Model (raw)**	FDCI	84%	64%	56%	68%	68%	82%
	Median	83.0 (3.3)	83.1 (8.5)	83.8 (5.9)	83.4 (4.4)	83.3 (6.0)	82.9 (3.5)
**3D Model (ComBat)**	FDCI	66%	78%	18%	32%	74%	68%
	Median	85.2 (4.2)	80.5 (9.3)	89.8 (4.2)	88.6 (3.8)	81.6 (7.0)	84.3 (5.5)
**2D Model (ComBat)**	Median	83.7 (3.8)	86.4 (5.9)	81.4 (8.5)	85.8 (4.4)	82.2 (6.5)	86.1 (5.4)

PPV = positive predictive value; NPV = negative predictive value;.

FDCI = frequency distribution comparison index (FDCI) value [(# of times 3D CNN > alternative model/total # of models)*100 for a percentage].

#### Feature visualization

**Figure 3:**
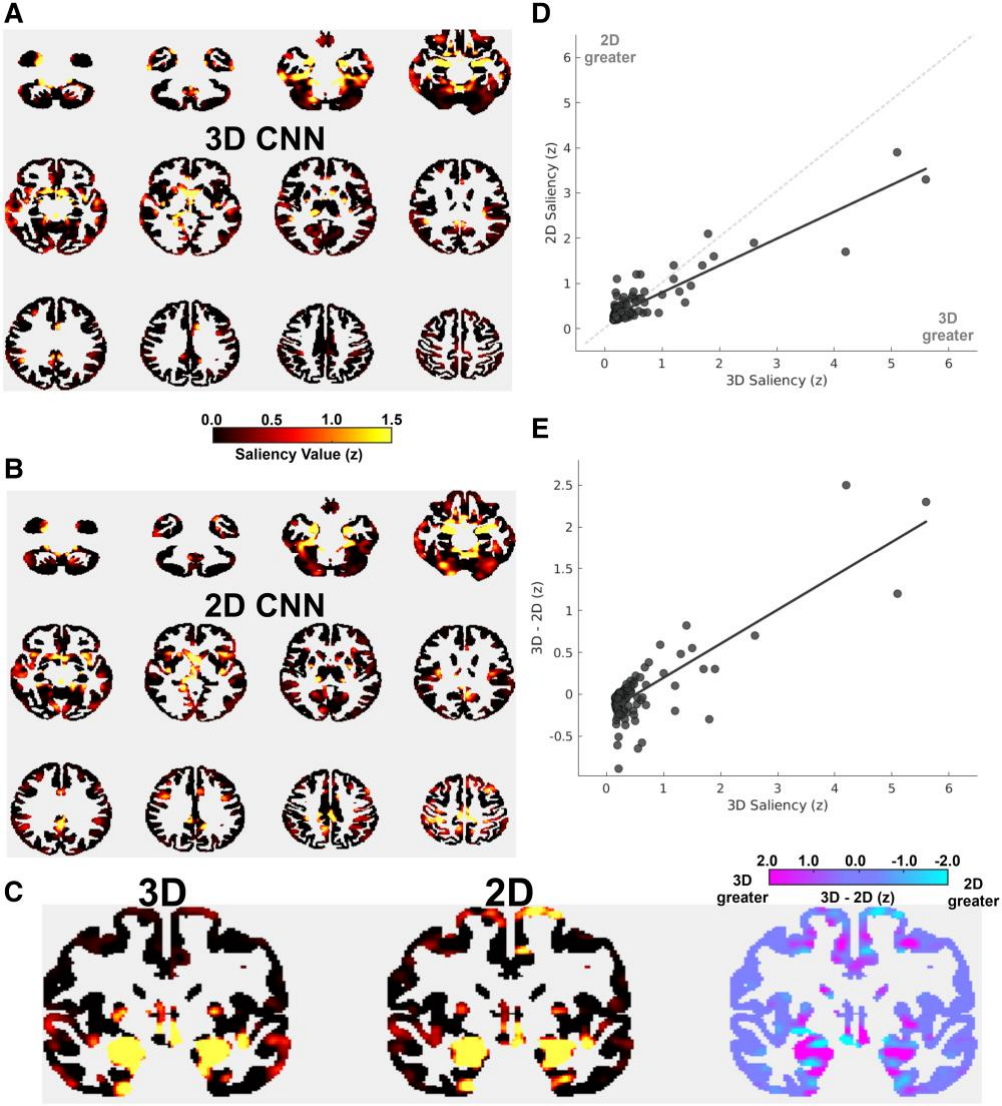
**Saliency maps. (A)** Axial slices of the saliency value at each voxel for the three-dimensional convolutional neural network (3D CNN), *z*-scored. **(B)** Axial slices of the saliency value at each voxel for the two-dimensional convolutional neural network (3D CNN), *z*-scored. **(C)** Representative coronal slice for the 3D CNN and 2D CNN saliency values, with the subtraction between the models on the far right, all in *z*-scores. (**D)** Scatterplot of saliency values from the AAL atlas between 2D and 3D models. The dashed grey line represents equal salience between models. The trend line is skewed downward, towards the 3D model having generally greater salience at higher values. A spearman’s rho run on this relationship was significant (r = 0.90, *P* < 0.0010). **(E)** Scatterplot of 3D saliency values with the subtraction of 3D and 2D model salience in the same ROI, demonstrating that the difference between 3D and 2D models tends to be highest in the most salient ROIs. A spearman’s rho run on this relationship was significant (*r* = 0.82, *P* < 0.001).

Figure 3A illustrates the regions that were found to be most salient by the 3D CNN model. We note that regions with the highest median weights were medial–ventral temporal (e.g. amygdala, hippocampus, and parahippocampal), cerebellar, and midline subcortical regions (e.g. thalamus and caudate). Figure 3B displays the saliency map for the 2D CNN, for which results were strongly similar to the 3D CNN. Figure 3C displays a coronal slice indicating that despite the regional similarity in importance, differences emerged, with 3D CNN saliency tending to be higher in medial–ventral temporal regions as well as midline structures, with regions of increased 2D CNN saliency more scattered and in non-temporal-limbic regions (e.g. precentral). Figure 3D quantifies the similarity, with a significant correlation (r = 0.90, *P* < 0.001) between ROI salience of the two models. However, it was noted that the trendline tilted in the direction of the 3D CNN axis, suggesting that salient regions tended to be of greater importance in the 3D CNN model. This qualitative observation was quantified in Fig. 3F, with a significant positive correlation (*r* = 0.82, *P* < 0.001) between 3D CNN ROI salience and the positive increase of 3D CNN above 2D CNN salience values.

### Single-subject performance

For demographic variables, we found no significant correlations with age (*r* = 0.013; *P* = 0.68) or group differences based on sex [*F*(1116) = 0.174; *P* = 0.68], but we found small but significant correlation effects, with individuals being less educated led to better model performance (*r* = −0.2; *P* < 0.001). Exploring this, we found that this relationship only held for TLE patients (*r* = −0.18; *P* < 0.001) but not HC (*r* = −0.093; *P* = 0.15). Exploring TLE-specific clinical variables, we found that performance did not differ for L- and R-TLE patients [*F*(493) = 0.252; *P* = 0.62], but was significantly affected by HS status [*F*(475) = 11.2; *P* < 0.001]. We note that group performance differed by ∼8%, with HS + (i.e. MRI-positive) patient accuracy at 90.2% and HS− (i.e. MRI-negative) patient accuracy at 82.7%. Finally, we found small but significant correlation values with clinical variables, with earlier age of onset (*r* = −0.16; *P* < 0.001) and longer duration (*r* = 0.17; *P* < 0.001) being associated with superior performance.

Due to the identified difference in performance of the 3D CNN based on MTS status, we explored this result further using post-hoc tests. First, we investigated how the 2D CNN performed on each subgroup, finding a performance of 86.4% for HS + (3.7% below the 3D CNN) and 80.4% for HS− (2.3% below the 3D CNN). Second, we investigated whether identified relationships between performance and patient characteristics were driven by mesial temporal sclerosis (MTS) status by investigating whether they remained consistent in the MRI- cohort (thus removing the effect of MRI+/MRI-). In this cohort, we found a small but significant correlation value with the age of onset (*r* = −0.17; *P* < 0.05), a trend with duration (*r* = 0.13; *P* = 0.067), and no significant relationship with education (*r* = −0.12; *P* > 0.05).

### Subsampled model evaluations

**Figure 4:**
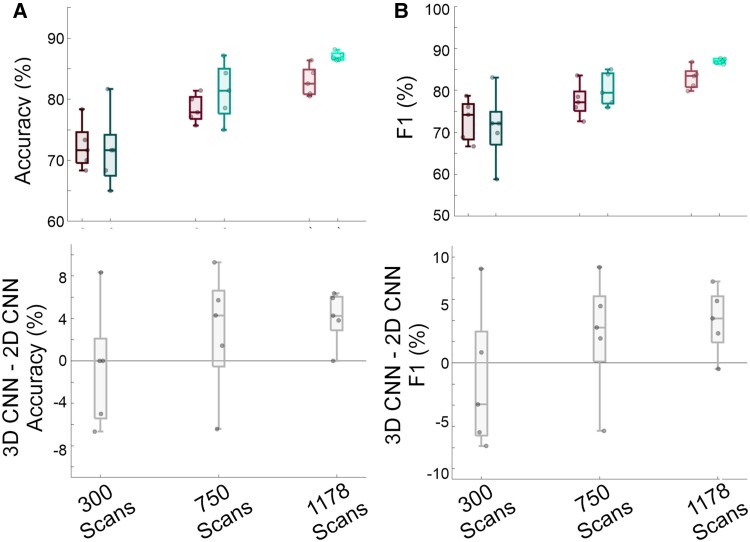
**Subsampled performance.** Each datapoint in the scatterplots represents one of the 5-folds from one of the 10 runs for each model (for a total of 50 datapoints). (**A**) Accuracy for the convolutional neural network (2D CNN; leftward boxes of couplets, transitioning from dark to light as sample increases) compared to the 3D CNN (righward boxes of couplets, transitioning from dark to light as sample increases; top) and subtraction plot across runs (bottom, grey represents subtractions, 3D CNN – 2D CNN). (**B**) *F*1-score for the convolutional neural network (2D CNN; compared to the 3D CNN and subtraction plot across runs (bottom, grey represents subtractions, 3D CNN – 2D CNN).

Figure 4 and Supplementary Table 4 display a sub-analysis in which 300, 750, and 1178 scans were run for 5-folds in the 3D CNN and 2D CNN models. We found that at 300 scans, median accuracy was lower, but similar for the 3D (71.7%) and 2D (71.7%) models. However, as more scans were added, the 3D model’s advantage over the 2D model became more apparent, with the full sample replicating our overall model evaluations, and the 3D model performing at 86.8% median accuracy and the 2D model at 82.6%.

### Harmonization: raw versus ComBat

The bottom rows of Table 2 contain the median values of harmonized model performance. First, we compared the 3D CNN on unharmonized and harmonized data. The 3D model with original data had an FDCI score on accuracy of 66% compared to the 3D CNN on harmonized data, an improvement for the 3D CNN original data model (binomial test: *P* < 0.05). Directly comparing accuracies via Wilcoxon sign-ranked test demonstrated only a trend towards the same pattern (*P* = 0.055). Overall, the 3D CNN on ComBat data had a median accuracy of 85.2% (IQR: 4.2%) and a median *F*1 score of 84.3% (IQR: 5.5%). Second, we compared the 2D CNN on unharmonized and harmonized data. The 2D model with original data had an FDCI score on accuracy of 40% compared to the 2D CNN on harmonized data, which was nonsignificant (binomial test: *P* > 0.05). Directly comparing accuracies via the Wilcoxon sign-ranked test similarly demonstrated no significant difference (*P* > 0.05). Overall, the 2D CNN on ComBat data had a median accuracy of 83.7% (IQR: 3.8%) and a median F1 score of 86.1% (IQR: 5.4%).

Finally, we compared the effect of 3D and 2D CNNs on harmonized data. The 3D model on harmonized data had an FDCI score on accuracy of 66% compared to the 2D CNN on harmonized data, an improvement for the 3D CNN harmonized data model (binomial test: *P* < 0.05). Directly comparing accuracies via the Wilcoxon sign-ranked test demonstrated the same relationship (*P* < 0.01).

## Discussion

The neural signature of TLE can be learned from standard T1-weighted imaging by a CNN, with detection improved by access to 3D spatial relationships. We show that a 3D CNN outperforms a 2D CNN and that this superiority is dependent on sample size, and this improvement is related to the increased focus on salient TLE regions. At our full sample size of 1178 MRI scans from 12 international surgical centres, the 3D CNN performed at a median accuracy of 86.4%. The diversity of our scans, both in geographical location range of imaging parameters, suggests that this result can be generalized and corroborates earlier reports of CNN performance in 2D CNNs at lower sample sizes.^27-30^ A widespread image harmonization method, ComBat,^38^ did not increase performance. Finally, we characterized how the model performed on an individual patient level to understand where improvements might be made. Critically, this model performed well on MRI-negative patients (accuracy at ∼82%), showing that the TLE neural signature is available to the CNN even when it is not detectable at a qualitative level by the human eye.

### Detecting the TLE signature using CNNs

Research has identified widespread patterns of structural pathology in patients with TLE, associated with disease characteristics^13,15,16^ and clinical outcomes.^17,18^ However, translation of these findings into clinical practice remains stymied by a myriad of factors, including questions about generalizability and how to best integrate into existing clinical practice. Pioneering tools such as NeuroQuant^19,20^ are useful, but in practice, the use of structural neuroimaging in the clinical setting and in machine-learning research remains mainly focused on feature selection.^21-26^ CNNs present a path towards incorporating the entire pattern of pathologies associated with TLE into clinical practice via both prediction and visualization.

Here, we found that a 3D CNN has a 35.3% accuracy improvement over chance, with a shuffled model correctly classifying ∼604 individuals and our 3D CNN model correctly classifying ∼1018 individuals. These results are in a cohort both larger and more heterogenous, and therefore more generalizable, than previous TLE CNN efforts which included a range from 157 to 359 scans. Visualizing model saliency, we confirmed that the 3D CNN model was leveraging the ‘TLE neural signature’, with a high salience in medial–ventral temporal, cerebellar, and midline subcortical regions. All these regions have been identified as structurally abnormal by imaging research in TLE.^10-13^ We note that while qualitative human diagnosis of TLE is focused on the hippocampus, a human clinician in collaboration with a tool based on CNN prediction and visualization output (i.e. ‘human in the loop’ AI) would have access to this much wider region of information.

But our performance of 86.4% accuracy leaves 160 individuals categorized incorrectly. To understand where the model struggled, we examined individual patient performance. Biological variables such as age and sex did not affect performance. Further, the model was equally accurate for both left and right TLE, important because despite elements of pathological asymmetry,^15^ the CNN learned consistent TLE patterns. But we observed small but statistically significant relationships between better model performance and patients with younger ages of seizure onset, longer duration of seizures, and lower education. Therefore, worse performance when patients had an older age of onset, shorter duration, and higher education. We believe the explanation for this pattern is interrelated. That is, this pattern is potentially due to the fact that in our cohort, patients with a lower age of seizure onset had longer durations, and a lower age of seizure onset is related to lower educational attainment.^44^ Further, a lower age of seizure onset (and therefore, longer duration and lower education) is related to the presence of human-detectable HS. Therefore, we cannot determine whether a longer duration leads to a stronger TLE signature (as would predicted by reports of accelerated cortical thinning^16,45^), whether an earlier age of seizure onset causes more developmental disruption (leading to a stronger TLE signature^15^), or whether this is all related to the presence of human-detectable HS (a marker of stronger TLE signature). We took a preliminary step in this investigation by seeing if the identified relationships were also present in the MRI-negative cohort (i.e. seeking to remove the question of MRI positivity and negativity). We found that the relationship between accuracy and age of onset was still significant, the relationship between accuracy and duration became a trend only, and the relationship with education was not significant. Future research that more fully disambiguates these possibilities is necessary.

As noted, a critical single-patient consideration in clinical decision-making is human-detectable HS (i.e. an MRI-positive patient).^4-6,8^ The absence of HS (i.e. MRI-negative patients^7,8^) in patients with TLE will typically relegate T1-weighted scans to a negligible impact on diagnosis and treatment. Here, as might be expected, the model had a statistically significant improvement in accuracy for MRI-positive (∼90% accuracy) compared to MRI-negative (∼82% accuracy) patients. But this 82% represents an improvement of >30% over our chance shuffled-CNN model. Given percentage of MRI-negative patients is increasing in epilepsy clinics,^46^ the ability to ‘rescue’ this magnitude of MRI-negative scans into a position where they can contribute to diagnosis and treatment planning would represent a significant clinical impact.

### Improving CNN performance: 3D CNN

More advanced models appear to increase CNN reliability. Our 3D CNN demonstrated a consistent improvement in accuracy over our 2D CNN. The magnitude of the improvement was modest (3.4%), so we investigated whether this was related to our sample size, as 3D CNNs require a large amount of data to avoid the problem of overfitting training data.^35^ Through subsampling, we found that at 300 scans there was no meaningful difference in performance between 3D and 2D CNN. It was only when we used the full available sample that superiority for the 3D CNN stabilized. The simple maxim ‘more is better’ is due to larger training sets allowing the model to avoid non-generalizable overfitting and therefore worse performance on the held-out test set. Because the 3D CNN is more interconnected, any benefits must be balanced against a larger danger of overfitting. Therefore, we believe that as TLE datasets continue to grow in size, the magnitude of 3D superiority will increase. At our sample size the superiority is emerging, but perhaps muted.

To understand what might underlie 3D CNN superiority, we compared model saliency maps to understand how the model’s behaviour changed in response to 3D information. We found that the TLE neural signature was nearly identical across models, with the correlation statistics equal to ∼0.90. The chief difference is that in regions which both models agreed were most salient, the 3D CNN saliency was greater. Further, in areas that both models found less salient, 3D CNN saliency was lower. Figure 3C illustrates this, in which medial and ventral regions which had the greatest salience had increased salience in the 3D model. In general, the 3D spatial relationships appear to let the 3D CNN more ‘confidently’ identify the TLE neural signature and disregard unimportant noise.

### Limitations and future directions

A key limitation of this study, and of any CNN that aspires to be generalizable, is the need for harmonization across disparate clinical environments, which can include different scanners, different sequences, and different patient populations. Here, we applied a batch-effect correction tool, ComBat,^38^ developed for genetics and widely applied in feature-based imaging studies, including TLE.^47^ We found that it did not improve 3D CNN performance, with some evidence towards an actual advantage for the original data. The 2D CNN performance was not significantly different based on harmonization. Finally, the 3D CNN advantage over 2D CNN was still present on the harmonized data, but the advantage appeared reduced relative to when using the unharmonized data. To explain the seeming reduction in 3D CNN performance on harmonized data, we note that while similar harmonization approaches applied to imaging have proven excellent for standard machine learning models as well as standard statistical approaches,^47^ they may remove biological information,^48^ thereby suggesting the need of alternative approaches for deep learning.^49,50^ An alternative explanation is that ComBat may have removed site effects which the 3D CNN model was identifying via subtle differences in cohorts across sites. Future harmonization work is needed to maximize CNN ability to detect the TLE neural signature, minimize noise, and determine more precisely which sources of information are being leveraged by more sophisticated models.

Further limitations include our incomplete clinical and demographic data. ‘Big’ data applications are often limited in their ability to be both wide and deep; here we chose wide due to our hypothesized sample size needs for a 3D CNN, confirmed in our subsampling analysis. Relatedly, our final weakness is the necessity to be ‘sure’ of an accurate TLE diagnosis.^27^ In our current sample, the diagnosis is based on the consensus of a multidisciplinary clinical team based on electrophysiology, structural data, semiology, and other clinical considerations. However, the gold standard is a patient with a seizure-free outcome following surgery. As data pooling in our field continues (e.g. ENIGMA-Epilepsy), seizure outcomes will be a critical variable to include moving forward. Further, in this cohort, we excluded bilateral TLE (BTLE) and extra TLE (ETLE). However, application of any diagnostic aid tool would occur before BTLE and ETLE had been ruled out, so future efforts can focus on understanding just how specific this neural pattern is to TLE,^29^ possibly in a cascaded manner (e.g. epilepsy versus control followed by localization of seizure onset zone). The combination of additional sources of information, such as sEEG, could provide additional opportunities to aid in lesion localization.

## Conclusion

Taken together, our findings highlight that the TLE neural signature can be detected on T1-weighted imaging by CNNs in a manner that could greatly benefit clinicians. By combining datasets to enlarge training samples, more advanced CNNs, in this case, an interconnected 3D CNN, can better identify regions of highest interest and improve performance. Methodological improvements such as improved harmonization and increasing data sizes are expected to further enhance CNN performance. The next steps will be to translate findings relating patient outcomes and their relationship to brain structure^17,18^ into CNNs.

## Supplementary Material

fcae346_Supplementary_Data

## Data Availability

Data from our work will be made available upon reasonable request.
