# Supplementary material for: Adding the third dimension: 3D convolutional neural network diagnosis of temporal lobe epilepsy
Source: Brain Commun. 2024 Oct 10;6(5):fcae346. doi: 10.1093/braincomms/fcae346 (PMC11520928; doi:10.1093/braincomms/fcae346)
Supplement: fcae346_Supplementary_Data [file fcae346_supplementary_data.docx]

**Supplementary Table 1. Variables Across Sites.**

| Site | TLE  *n* | HC  *n* | Age  *Mean (SD)* | Sex  *(% female)* | Side  *(% left)* | HS  (% yes) |
| --- | --- | --- | --- | --- | --- | --- |
| Bonn | 42 | 32 | 40.7 (12.8) | 53.4 | 69.0 | 88 |
| Emory | 89 | 58 | 38.0 (12.8) | 58.9 | 42.7 | 52 |
| Liverpool | 24 | 0 | 32.0 (11.5) | 62.5 | 62.5 | 29 |
| MUSC | 147 | 56 | 42.0 (13.1) | 62.7 | 51.0 | 48 |
| Northwell | 10 | 6 | 35.3 (10.3) | 60.0 | 50.0 | 10 |
| NYU | 27 | 15 | 32.7 (10.7) | 64.3 | 48.1 | 63 |
| Penn | 55 | 10 | 37.9 (11.5) | 42.2 | 21.8 | 30 |
| Pitt | 31 | 19 | 39.1 (13.1) | 66.0 | 41.9 | 34 |
| Rush | 52 | 0 | 35.4 (10.8) | 58.8 | 48.1 | 61 |
| UCSD | 74 | 83 | 37.3 (13.2) | 59.9 | 45.9 | 55 |
| UCSF | 38 | 0 | 31.2 (12.0) | 55.3 | 57.9 | 32 |
| HCP | 0 | 310 | 36.6 (14.8) | 49.7 | - | - |

**Supplementary Table 2. Imaging Parameters across sites.**

|  | **Bonn** | **MUSC** | **Northwell** | **Emory** | **NYU** | **Upenn** | **Upitt** | **Liverpool** | **UCSD 1.5T** | **UCSD 3T** | **UCSF** | **Rush** |
| --- | --- | --- | --- | --- | --- | --- | --- | --- | --- | --- | --- | --- |
| Scanner | Magnetom | Verio, Skyra | Prisma | Skyra | Prisma | Prisma Fit | Prisma | GE | GE SIGNA | GE Discover | GE Discover 750 | Siemens Verio |
| Field | 3T | 3T | 3T | 3T | 3T | 3 T | 3T | 3T | 1.5T | 3T | 3T | 3T |
| Software version |  | VE11c | VE11b |  | VE11 | VE11c | VE11c |  | 14.0_M5_0737.f | DV22.0_V02_1122.a | DV26.0_R01_1725.a | syngo MR B19 |
| Sequence | MPRAGE | MPRAGE | MPRAGE | MPRAGE | MPRAGE | MPRAGE | MPRAGE | FSPGR | MPRAGE | FSPGR | FSPGR | MPRAGE |
| Orientation | Sagittal | Sagittal | Sagittal | Transversal | Sagittal | Sagittal | Sagittal | Sagittal | Sagittal | Sagittal | Sagittal | Sagittal |
| TR (ms) | 650 | 1900-2250 | 1900 | 2200 | 2100 | 1900-2400 | 1900-1960 | 820 | 1073 | 808 | 808 | 2300 |
| TE (ms) | 3.87 | 2.36-4.18 | 2.36 | 2.48 | 2.72-2.75 | 2.24-2.84 | 2.36-3.77 | 3.22 | 4.87 | 3.16 | 3.16 | 3.29 |
| TI (ms) | 650 | 900-925 | 900 | 900 | 900-1003 | 900-1006 | 900 | 450 | 1000 | 640 | 600 | 900 |
| FA (deg) | 10 | 9 | 9 | 8 | 8-12 | 8-9 | 9-12 | 12 | 8 | 8 | 8 | 8 |
| FOV (mm) | 256 | 256 | 256 | 230 | 256 | 256 | 256 | 256 | 256 | 256 | 256 | 256 |
| VS (mm) | 1x1x1 | 1x1x1 | 1x1x1 | 0.9x0.9x0.9 | 1x1x1 | 1x1x1 | 1x1x1 | 1x1x1 | 1x1x1 | 1x1x1 | 1x1x1 | 0.6x0.6x0.6 |

**Supplementary Table 3. Hyperparameters**

| **Hyperparameter** | **Values** |
| --- | --- |
| Learning rate | 0.00001, 0.0001, 0.001, 0.01, 0.1 |
| Optimizer | SGD and Adam |
| Input channel size at each layer | "16,32,64,128,256", "32,64,64,128,128", "32,64,128,256,512", "64,64,128,128,256", "64,128,128,256,512", "128,128,128,256,256", "128,128,128,256,512" |
| Batch size | 8, 16 |

**Supplementary Table 4. Subsampled Performance**

|  | **300 scans**  **(150 TLE / 150 HC)** | | **750 scans**  **(375 TLE / 375 HC)** | | **1178 scans**  **(569 TLE / 569 HC)** | |
| --- | --- | --- | --- | --- | --- | --- |
|  | 3D | 2D | 3D | 2D | 3D | 2D |
| **Accuracy** | 71.7 (6.7) | 71.7 (5.0) | 81.4 (7.3) | 77.9 (3.6) | 86.8 (1.1) | 82.6 (4.0) |
| **Sensitivity** | 66.7 (18.1) | 72.7 (14.4) | 75.6 (8.7) | 76.6 (8.9) | 85.5 (4.9) | 83.9 (11.4) |
| **Specificity** | 77.8 (13.4) | 70.6 (23.5) | 93.4 (20.4) | 78.2 (6.3) | 88.0 (4.4) | 83.8 (10.8) |
| **PPV** | 78.6 (21.6) | 71.1 (20.6) | 89.1 (14.6) | 76.1 (11.2) | 87.7 (3.3) | 84.7 (7.3) |
| **NPV** | 68.6 (13.9) | 72.7 (7.7) | 77.7 (8.9) | 78.3 (3.8) | 86.0 (3.8) | 82.7 (8.3) |
| **F1** | 72.1 (7.8) | 74.2 (8.4) | 79.5 (7.3) | 77.1 (4.6) | 87.0 (1.0) | 83.4 (3.7) |
